# Determinants of Homelessness (SODH) in North West England in 2020

**DOI:** 10.1093/eurpub/ckac130.122

**Published:** 2022-10-25

**Authors:** M Mabhala

**Affiliations:** University of Chester, Chester, UK

## Abstract

**Background:**

Poverty creates social conditions that increase the likelihood of homelessness. These include exposure to traumatic life experiences; social disadvantages such as poor educational experiences; being raised in a broken family, care homes or foster care; physical, emotional, and sexual abuse; and neglect at an early age. These conditions reduce people's ability to negotiate through life challenges.

**Methods:**

This cross-sectional study documents the clustering and frequency of adverse social conditions among 152 homeless people from four cities in North West England between January and August 2020.

**Results:**

Two-step cluster analysis showed that having parents with a criminal record, care history, and child neglect/abuse history was predictive of homelessness. The cluster of indicator variables among homeless people included sexual abuse (χ2 (N = 152) = 220.684, p < 0.001, Cramer's V = 0.7), inappropriate sexual behaviour (χ2 (N = 152) = 207.737, p < 0.001, Cramer's V = 0.7), emotional neglect (χ2 (N = 152) = 181.671, p < 0.001, Cramer's V = 0.7), physical abuse by step-parent (χ2 (N = 152) = 195.882, p < 0.001, Cramer's V = 0.8), and physical neglect (χ2 (N = 152) = 205.632, p < 0.001, Cramer's V = 0.8).

**Conclusions:**

Poverty and homelessness are intertwined because of the high prevalence of poverty among the homeless. Poverty sets up a chain of interactions between social conditions that increase the likelihood of unfavourable outcomes: homelessness is at the end of the interaction chain. Interventions supporting families to rise out of poverty may also reduce entry into homelessness.

**Key messages:**

- Being poor is associated with so many childhood adversities that it may be considered an ACE in itself.
- Reducing poverty might be one strategy to reduce both ACEs and homelessness.

